# Machine-learning based control of bi-modular multilevel PWM inverter for high power applications

**DOI:** 10.1371/journal.pone.0305759

**Published:** 2024-07-22

**Authors:** Ravi Teja Srungaram, Kishore Yadlapati

**Affiliations:** 1 Department of Electrical and Electronics Engineering, Jawaharlal Nehru Technological University Kakinada, Kakinada, Andhra Pradesh, India; 2 Department of Electrical and Electronics Engineering, JNTUK University College of Engineering Narasaraopet, Narasaraopet, Andhra Pradesh, India; University of Cagliari, ITALY

## Abstract

This paper presents the topology and machine learning-based intelligent control of high-power PV inverter for maximum power extraction and optimal energy utilization. Modular converters with reduced components economic and reliable for high power applications. The proposed integrated intelligent machine learning based control delivers power conversion control with maximum power extraction and supervisory control for optimal load demand control. The topology of the inverter, operating modes, power control and supervisory control aspects are presented. Simulation is carried out in MATLAB/SIMULINK to verify the feasibility of the proposed inverter and control algorithm. The experimental study is presented to validate the simulation results. The operational performance of the proposed topology is evaluated in terms of operational parameters such as regulation of output power, and load relay control and is compared to existing topologies. The economic performance is also evaluated in terms of power switch sizing and reliability in power delivery concerning switch or power sources failure.

## 1. Introduction

Large scale photovoltaic (PV) integration to grid and PV assisted electric drives demand for optimal energy consumption for efficient usage of renewable PV sources. The state-of-art converters for PV integration and PV-assisted drives is provided in this section. Also, machine learning algorithms for PV energy estimation and load parameter estimation is reviewed. The recent multilevel inverter topologies [1, 2] for PV integration to grid provide single stage conversion, fault tolerance, unbalanced operation. Modularity in these multilevel converters is finding a growing interest owing to power sharing [3, 4], distribution of maximum power point (MPP) tracking control [5, 6] for independent control and reliability. Topologies with Wave shaping followed by dc-ac stage [6] where sub modules re employed for wave shaping. Parallel connected modules control [7], model predictive control for reduced calculation burden [8], modular converter with flying-capacitor like properties [9], flying-capacitor model with reduced number of components [10], asymmetrical multilevel inverter topologies [11, 12], inherent voltage balancing capability modules [13] made modular converters feasible to PV-Grid integration. The merit of modular converters is also validated for variable frequency drives [14, 15], power quality enhancement [16], front end rectification [17], energy storage [18]. These topologies suffered from increased control complexity, high component count and de-rating of the power switches due to sub-module structures. In parallel, various unconventional topologies were implemented to minimize the component count. Nine different reduced switch topologies were presented in [19] for drives and renewable energy integration. Symmetric and asymmetric staircase cascading multilevel inverter topologies were presented in [16, 20]. The performance is evaluated in terms of power distribution among modules, blocking voltage, number of switches, switching losses compared to conventional topologies. Switching capacitor topologies also showed good performance for DC-AC conversion [21]. However, these unconventional topologies require specially synthesized control structures and are prone to reliability considerations. The Reliability study [22, 23] and efficient component sizing [24] are key commercial power conversion aspects. In this context, high-power PV integration needs multilevel inverters with modularity and fewer components for reliable operation and semi-conductor footprint reduction [25, 26]. Utilization of heuristic and artificial intelligent techniques for power control in renewable energy systems integrated to grid was reported [27, 28] in which atom search optimization, fuzzy logic-based control, predictive control was adopted for efficient and robust control.

Various machine learning applications are developed which include policy approximations for energy management in PV systems [29], energy loss assessment foe PV sizing and ensemble learning for fault detection in PV systems [30], machine learning for PV energy forecasting [3], clustering and deep learning algorithms for energy estimation [31], Adaline neural networks [32], energy management systems [33] for improved forecasting methods for capacity firming. All these technologies and methodologies involve multiple-stage conversion, complex algorithms, and convergence.

Therefore, the present work attempts to combine the reliability merit of modularity with the reduced component count. A bi-modular multilevel inverter is developed which could provide any level voltage between seven and nineteen with the semi-conductor switch component count equal to that of conventional five level inverters. Also, machine learning-based supervisory control for PV power estimation and corresponding load demand control is presented.

The rest of the paper is organized as follows. Section 2 presents the switching logic and control of the inverter. Section 3 presents control algorithm. Section 4 presents simulation study of the proposed topology. Section 5 presents the experimental validation of inverter performance. Section 6 presents conclusions.

## 2. Bi-modular multilevel inverter

**Fig 1:**
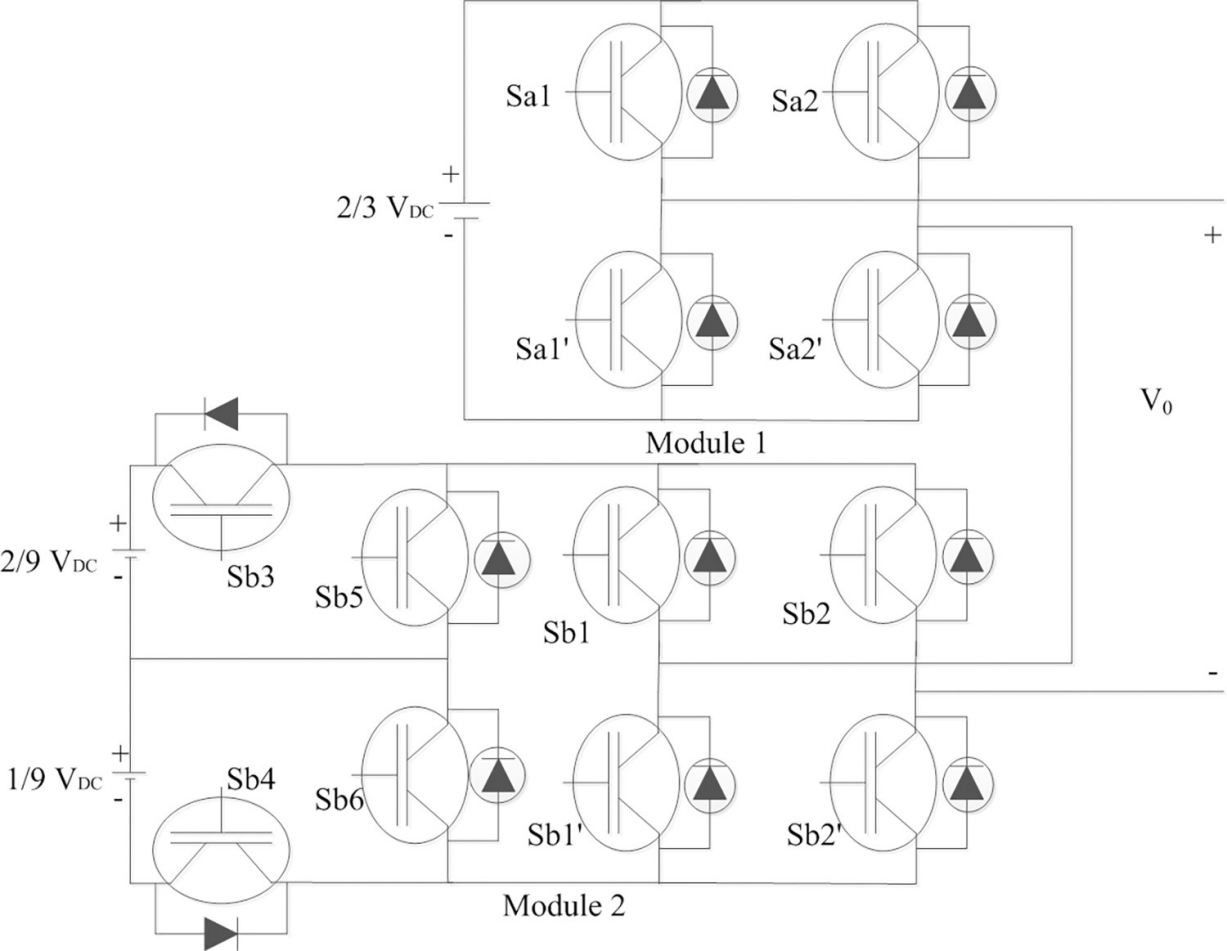
Bi-modular multilevel inverter topology.

The schematic of the modular multilevel inverter is illustrated in Fig 1. DC voltages are in the ratio 1:2:6, respectively [34] as shown in Fig 1. Each module has two functions viz. voltage level generation and polarity generation. The H-Bridge in the modules generate polarity. Asymmetrical switches in module 2 (Sb3, Sb4, Sb5, Sb6) generate three different voltage levels. The cascaded modules therefore generate a respective voltage level as the algebraic sum of voltage of the two modules.

### 2.1 Switching combinations for output voltage levels generation

**Fig 2:**
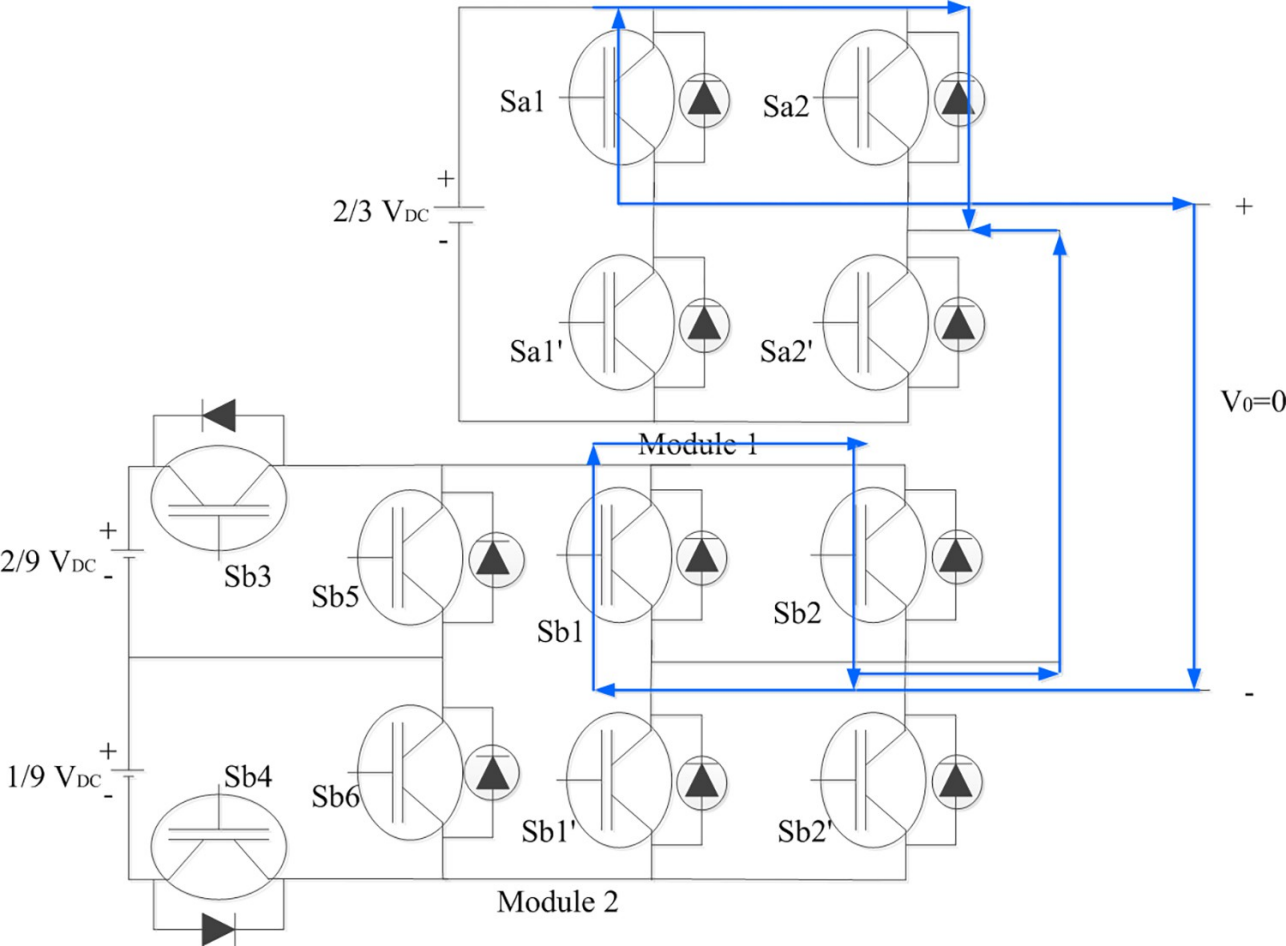
Switching combinations for generation of zero output voltage level.

The switching combinations to achieve each level in positive half cycle of output voltage is shown in Fig 2. The switches turned ON in respective module were identified along with the flow of current in each switch and load. Each module’s contribution to generating respective voltage levels is shown in each case. The similar combinations with complementary switches in each module would develop voltage levels for negative half cycle of the output voltage.

#### 2.1.1. To generate V_0_ = 0

Top switches in H-bridges in both modules are turned ON so that sources are not connected to load. Each module generates zero voltage which accounts for zero output voltage as shown in Fig 2.

#### 2.1.2. To generate V_0_ = +(1/9) V_DC_, +(2/9) V_DC,_ +(3/9) V_DC_

**Fig 3:**
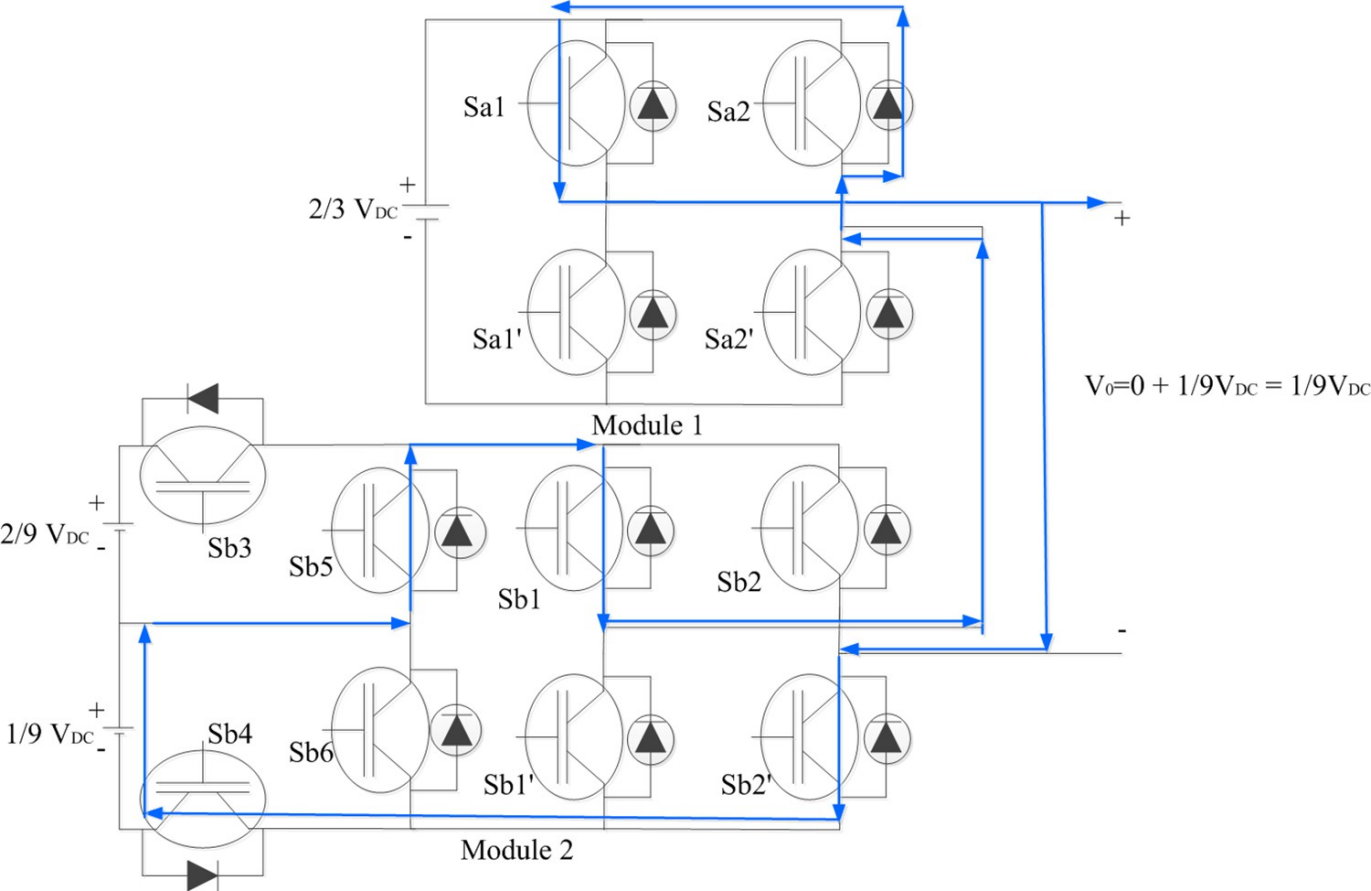
Switching combinations for generation of +1/9 V_DC_ output voltage level.

Top switches in Module 1 H-bridge are turned ON so that it generates zero voltage. Sb4 and Sb5 of Module 2 asymmetrical bridge connects 1/9 V_DC_ to its H-bridge. With Sb1 and Sb2^’^ turned ON + (1/9) V_DC_ is generated across Module 2. Thus, the total output voltage level is algebraic sum of voltage levels of two modules which accounts for + (1/9) V_DC_ as shown in Fig 3. Similarly, only Module 2 corresponds to generation of next two voltage levels with connection of 2/9 V_DC_ alone and combination of 1/9 V_DC_ and 2/9 V_DC_ to Module 2 H-bridge respectively with corresponding asymmetric switches turned ON.

#### 2.1.3. To generate V_0_ = +(4/9) V_DC_, +(5/9) V_DC,_ +(6/9) V_DC_

**Fig 4:**
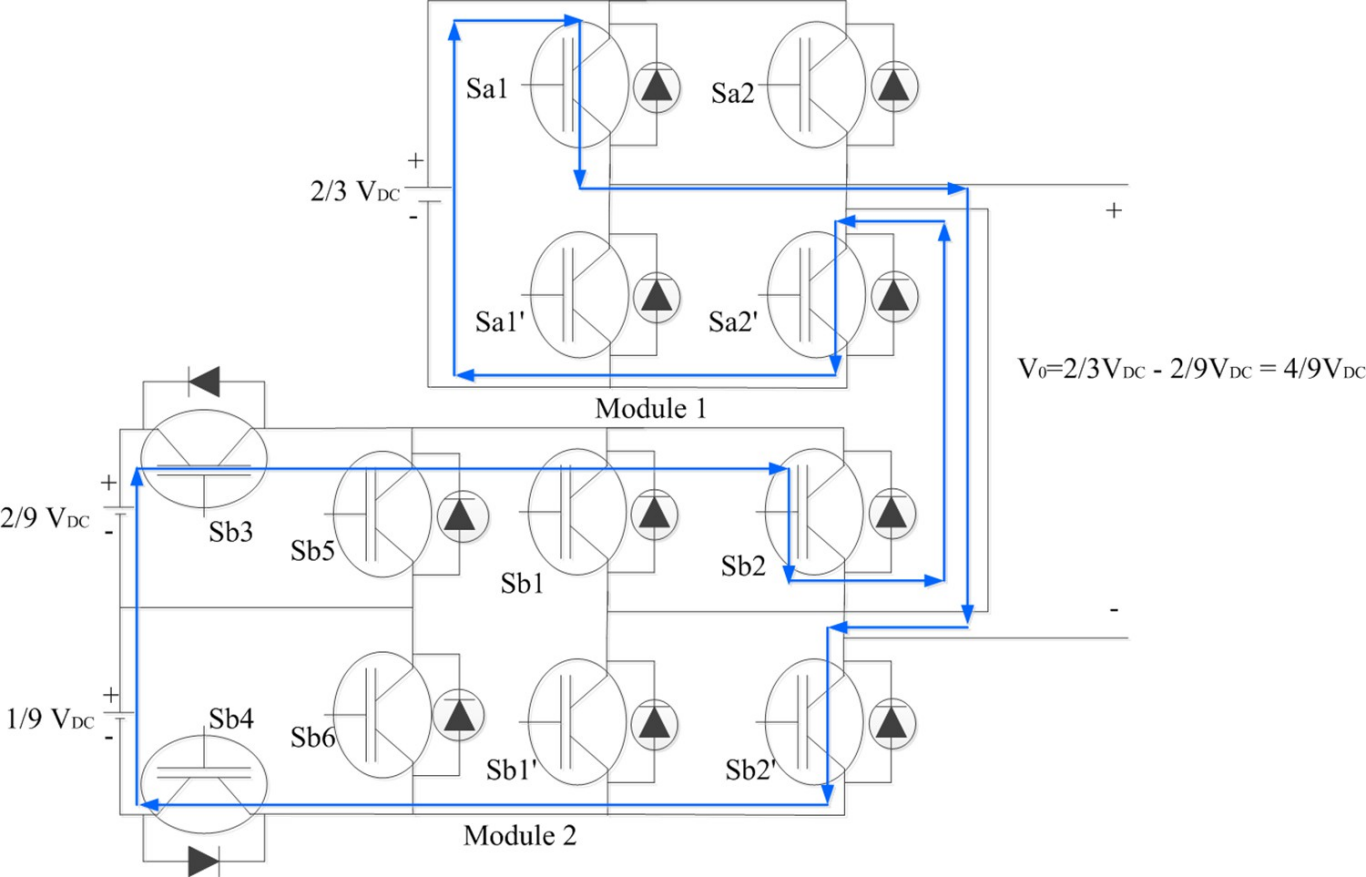
Switching combinations for generation of +4/9 V_DC_ output voltage level.

Sa1 and Sa2’ of Module 1 H-Bridge connects 2/3 V_DC_ to its output. Sb3 and Sb4 of Module 2 asymmetrical bridge connects 2/9 V_DC_ to its H-bridge. With Sb2 and Sb1^’^ turned ON—(2/9) V_DC_ is generated across Module 2. Thus, the total output voltage level is algebraic sum of voltage levels of two modules which accounts for + (2/3) V_DC_ + (-2/9) V_DC_ = +(4/9) V_DC_ as shown in Fig 4. Similarly, only Module 2 corresponds to generation of next two voltage levels with connection of -1/9 V_DC_ alone and combination of -3/9 V_DC_ to Module 2 H-bridge respectively with corresponding asymmetric switches turned ON respectively.

#### 2.1.4. To generate V_0_ = +(7/9) V_DC_, +(8/9) V_DC,_ + V_DC_

**Fig 5:**
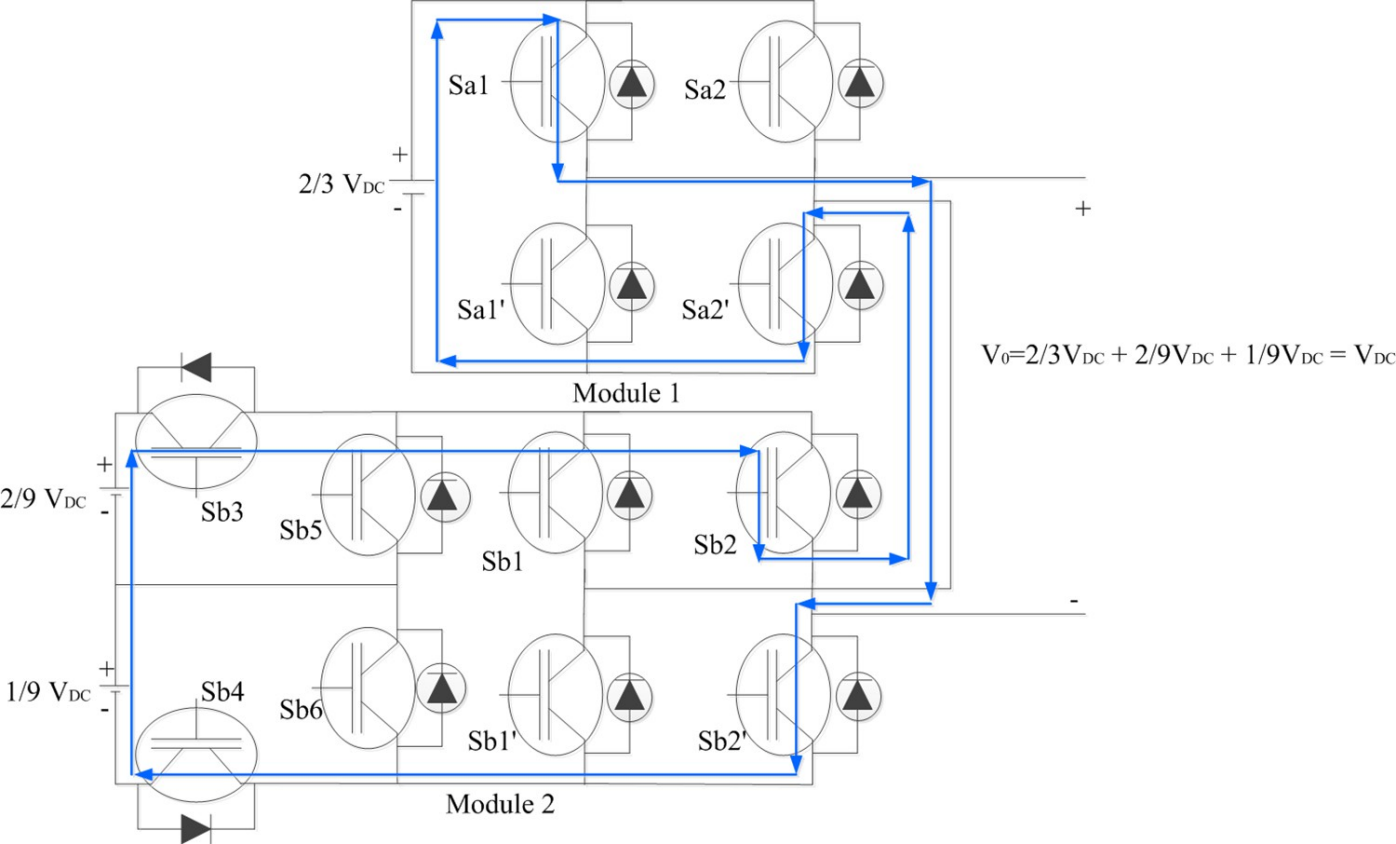
Switching combinations for generation of +V_DC_ output voltage level.

Sa1 and Sa2’ of Module 1 H-Bridge connects 2/3 V_DC_ to its output. Sb3 and Sb4 of Module 2 asymmetrical bridge connects 2/9 V_DC_ to its H-bridge. With Sb1 and Sb2^’^ turned ON + (3/9) V_DC_ is generated across Module 2. Thus, the total output voltage level is algebraic sum of voltage levels of two modules which accounts for + (2/3) V_DC_ + (3/9) V_DC_ = + V_DC_ as shown in Fig 5. Similarly, only Module 2 corresponds to generation of voltage levels with connection of +2/9 V_DC_ alone and +1/9 V_DC_ to Module 2 H-bridge respectively with corresponding asymmetric switches turned ON generate +7/9 V_DC_ and + 8/9 V_DC_.

### 2.2 Pulse width modulation and output voltage

**Fig 6:**
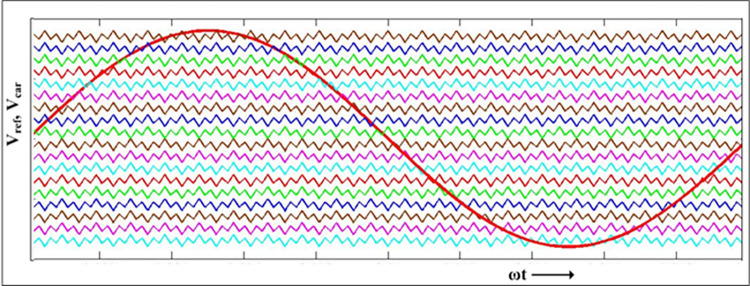
Reference signal and level shifted modified triangular carrier for the first stage PWM signals generation.

The output frequency sinusoidal reference signal is compared to level shifted modified triangular carrier signals as shown in Fig 6. An asymmetrical carrier width is produced to improve the switching behavior, increasing the apparent switching frequency.

The expression for unit template of carrier waveform pertaining to k^th^ switching period is given as

$$V_{C}(t)=\{\begin{array}{c} -1+\frac{4}{9}(t-kT),\;(k)T<t\leq (k+\frac{1}{6})T\\ \frac{-25}{27}-\frac{2}{9}(t-(k+\frac{1}{6})T,\;(k+\frac{1}{6})T<t\leq (k+\frac{1}{3})T)\\ \frac{-26}{27}+\frac{4}{9}(t-(k+\frac{1}{3})T),\;(k+\frac{1}{3})T<t\leq (k+\frac{1}{2})T\\ \frac{-8}{9}-\frac{4}{9}(t-(k+\frac{1}{2})T),\;(k+\frac{1}{2})T<t\leq (k+\frac{2}{3})T\\ \frac{-26}{27}+\frac{2}{9}(t-(k+\frac{2}{3})T),\;(k+\frac{2}{3})T<t\leq (k+\frac{5}{6})T\\ \frac{-25}{27}-\frac{4}{9}(t-(k+\frac{5}{6})T),\;(k+\frac{5}{6})T<t\leq (k+1)T \end{array}\}\;\mathrm{for}\;k=0,1,2,3\ldots ..\infty$$ (1)

Where, *T* is period for one switching cycle with $\omega_{s}=\frac{2\pi }{T}$ is the angular switching frequency.

Then, the level shifted carrier waveforms are given as

$$V_{(x-9)c}(t)=\frac{x}{9}+V_{c}(t)$$ (2)

for x = 0, 1, 2, …..18 represent the eighteen level shifted carrier waveforms.

The reference sinusoidal signal is given as

$$V_{r}(t)=M\;sin\;(\omega_{r}t)$$ (3)

With |*M*|≤1 and *ω*_*r*_ is the reference angular frequency for the output voltage waveform.

The following implicit relations represent the switching instants in each switching period for different carrier waveforms with natural sampling.


$$A_{(x-9)k}^{a}=kT+\frac{T}{4}\left(1+V_{r}(A_{(x-9)k}^{a})\right)$$ (4)

$$B_{(x-9)k}^{a}=kT+\frac{T}{4}\left(3-V_{r}(B_{(x-9)k}^{a})\right)$$ (5)

Where, $A_{(x-9)k}^{a}$, and $B_{(x-9)k}^{a}$ are leading and trailing switching instants of x^th^ carrier in k^th^ switching period.

The pulse width modulated (PWM) signal generates two states viz. 1 and 0 respectively for reference wave above and below carrier wave which is given as follows

$$V_{(x-9)a}(t)=\left\{\begin{array}{l} +1,\;A_{(x-9)k}^{a}\leq t\leq B_{(x-9)k}^{a}\\ \;0,\;otherwise \end{array}\right\}$$ (6)

Then, the output voltage is the algebraic sum of pulse width modulated waveforms develop by each carrier raised by a factor of *MV*_*DC*_. Thus, the output voltage

$$V_{0}(t)=MV_{DC}\sum_{x=0}^{18}V_{(x-9)a}(t)$$ (7)

whose Fourier series representation is given as

$$V_{0}(t)=MV_{DC}\;\sin\left(\omega_{r}t\right)+MV_{DC}\sum_{m=0}^{\infty }\sum_{n=-\infty }^{\infty }V_{mn}sin(m\omega_{s}t+n\omega_{r}t)$$ (8)

The PWM first stage PWM patterns obtained from Eq (6) are designated as 1 to 9 and -1 o -9 in Table 1. Now, from the operational modes explained from Fig 2 the contribution of each switch to different output voltage levels is obtained. Thus, the gating signals for each switch are obtained by logical OR operations of all such PWM patterns for which the respective switch contribution is required.

**Table 1:** Gating pulse combinations.

Gating pulses for Switches	Logic OR combination
S_a1_	1,2,3,4,5,6,7,8,9,-1,-2,-3
S_a2_	1,2,3,-1,-2,-3,-4,-5,-6,-7,-8,-9
S_b1_	1,2,3,6,7,8,9,-4,-5
S_b2_	4,5,6,-1,-2,-3,-7,-8,-9
S_b3_	2,3,4,8,9,-2,-3,-4,-8,-9
S_b4_	1,3,5,7,9,-1,-3,-5,-7,-9
S_b5_	1,5,7,-1,-5,-7
S_b6_	2,4,8,-2,-4,-8

## 3. Machine learning based control of converter

Machine learning based control is utilized for power flow control and optimal load scheduling. The control objectives include extracting maximum power corresponding to irradiance at any given instance, delivery of power to load at regulated voltage, and estimation of PV power for succeeding time intervals for time shifting of allowable loads for optimization of PV energy consumption.

### 3.1 Reinforcement learning ANN training for MPP and PV power estimation

**Fig 7:**
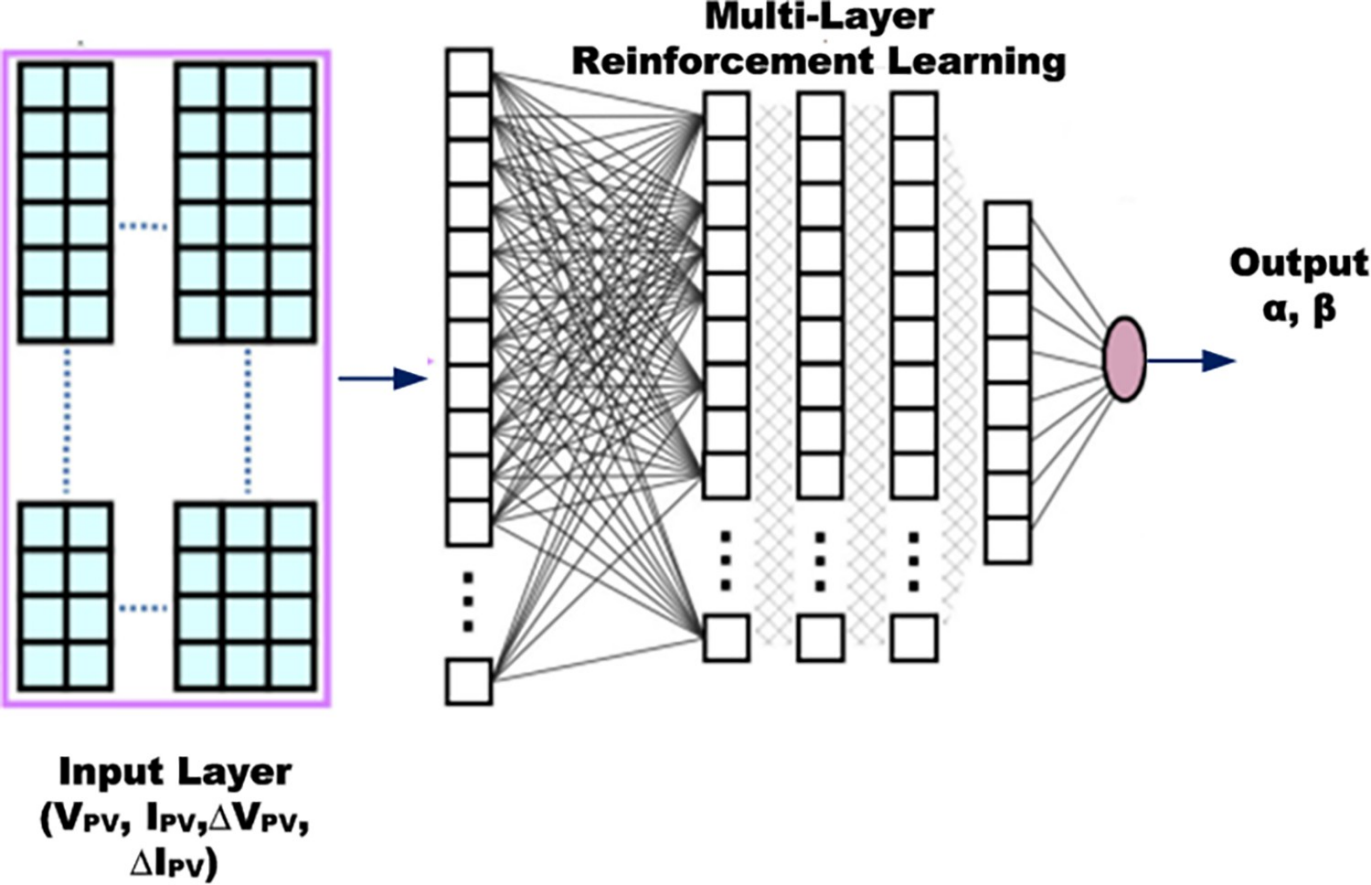
Machine learning scheme for MPP and PV power estimation.

Multi-layer reinforcement learning artificial neural network (ANN) is employed for pattern recognition type machine learning to estimate MPP and hourly PV power due to its ability to recognize various patterns and cluster them for provided input-output combination. This is highly suitable for varied conditions of irradiance under different days of different seasons to produce large variety of power variance combinations. The scheme of machine learning is shown in Fig 7 which consists of ΔP_PV_ (i.e. P_PV_(k)–P_PV_(k-1)) as input with multiple reinforcement training middle layers and the acceleration factor α as the output.

**Fig 8:**
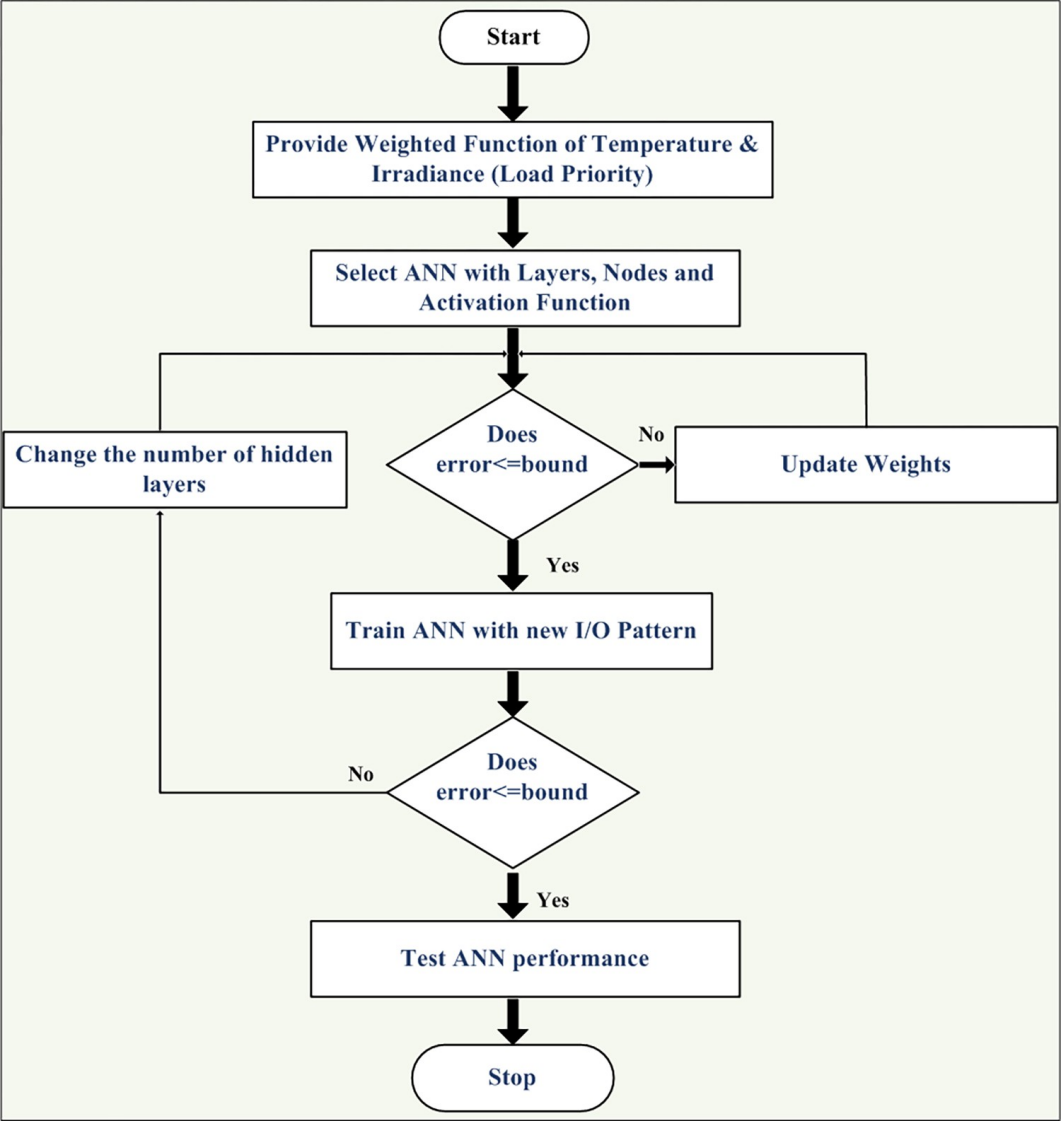
Machine learning algorithm for integrated intelligent control.

The detailed training algorithm in shown in Fig 8 in which the input data samples are clustered in to subsets in various epoch which undergo several iterations until the error in power estimation reaches threshold value.

**Fig 9:**
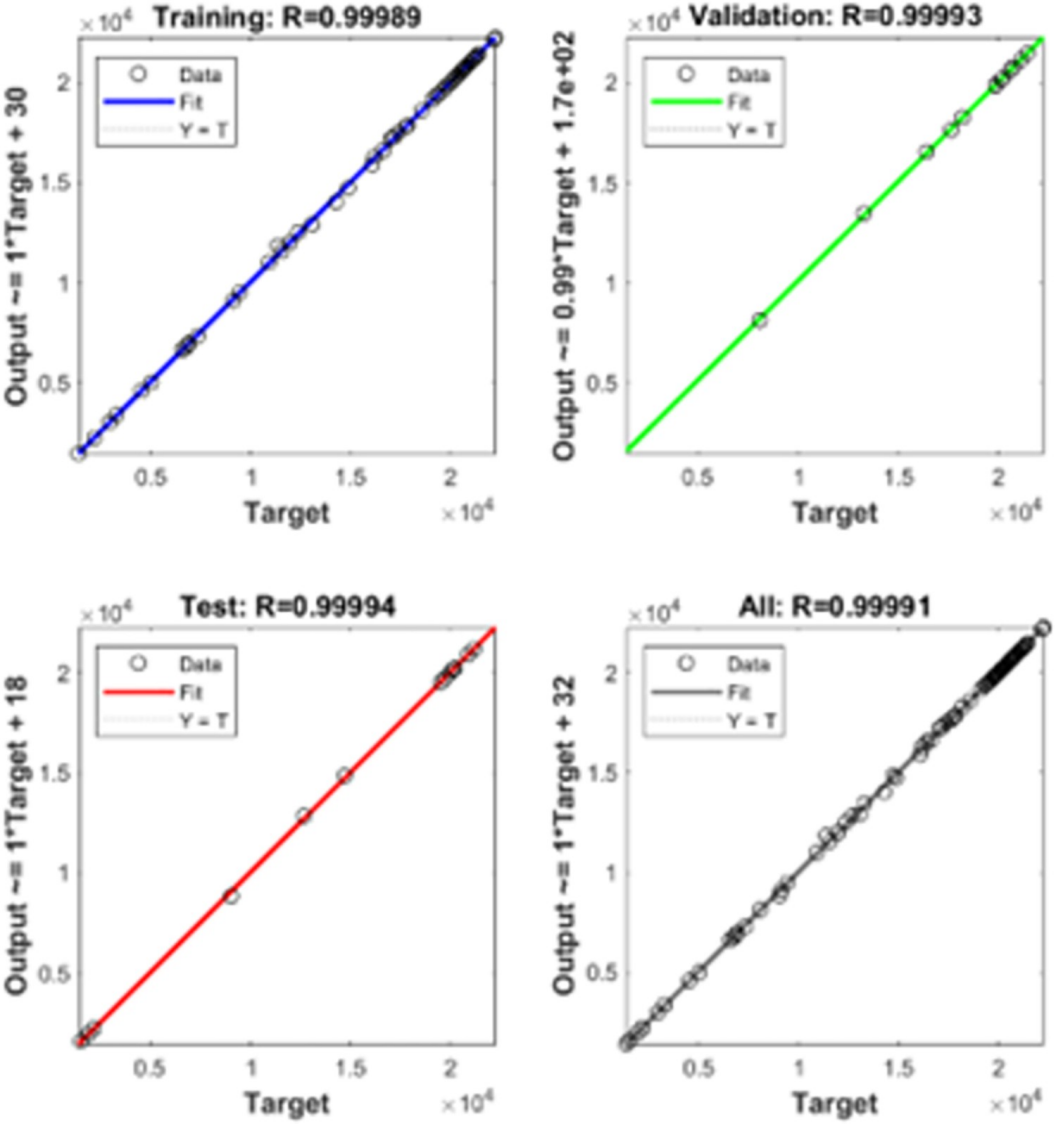
Iterations of machine learning.

**Fig 10:**
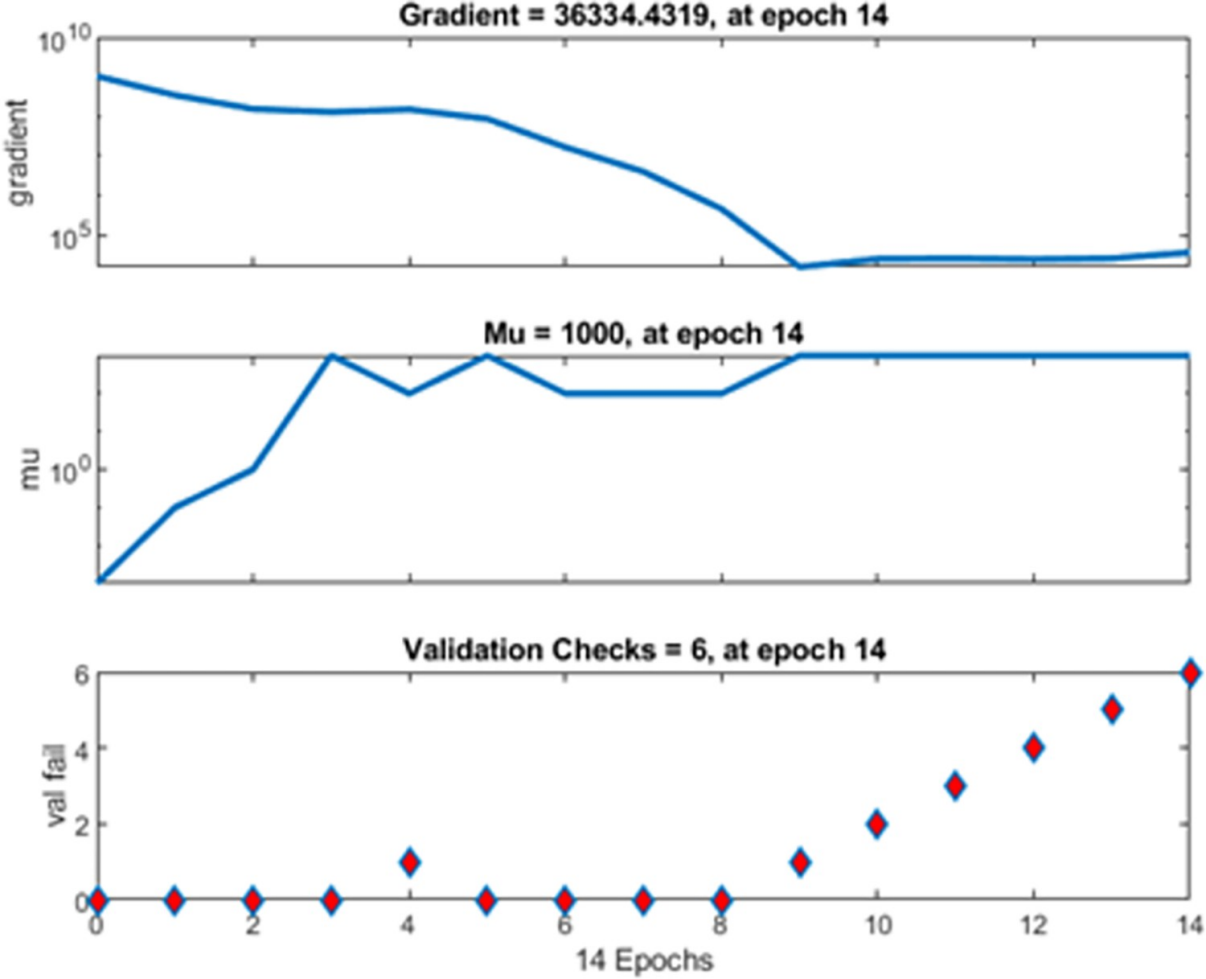
Gradient of mean squared error and validation checks.

**Fig 11:**
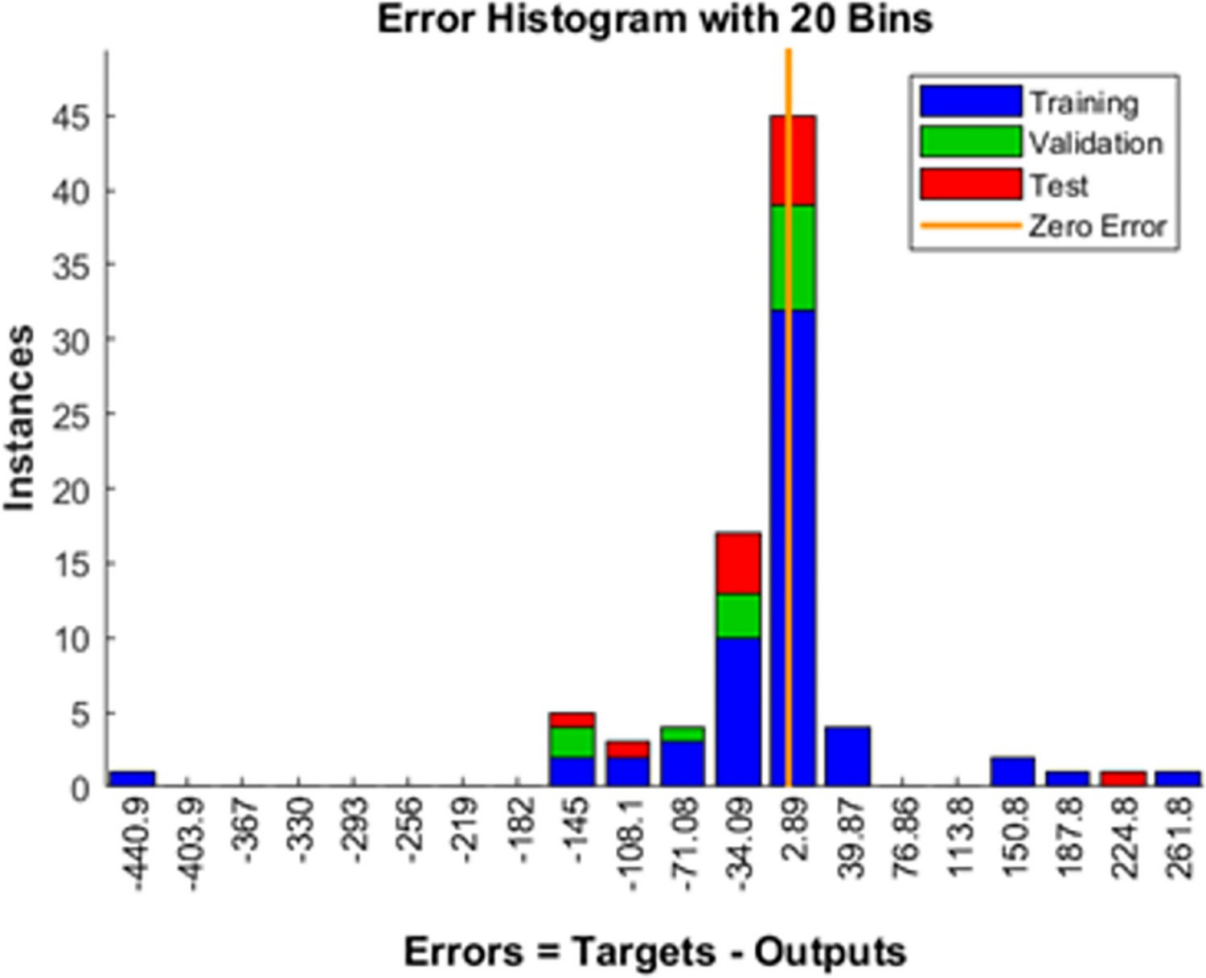
Error histogram.

The training sets as shown in Table 2 is utilized for machine learning. The completed data utilized for training is provided in S1–S4 Files. The training of multi-layer ANN for MPP tracking and load demand control is depicted in Figs 9 and 10. The convergence was obtained at eighth epoch of training. The regression for training, validation and training data sets was observed in Fig 9 which depicts accuracy to of the training to target output data. The gradient of mean of squared error and validation checks were shown in Fig 10 which also depict the convergence of the neural network. The validation of estimation is shown in Fig 11 in which larger set of instances proved closeness to zero error.

**Table 2:** Sample training data.

Time Interval (HH:MM)	Irradiance (W/m^2^)	Temperature (°C)	ΔP_PV_	α	Power Generation (pu)
13:00	504	41	5	+0.09	0.2539472593
13:05	465	41	7	+0.093	0.2313561481
13:10	465	41	8	+0.097	0.2313561481
13:15	487	41	-5	-0.084	0.2440998519
13:20	569	42	4	+0.076	0.2892241481
13:25	505	42	-6	-0.079	0.2521515556
13:30	474	42	3	+0.034	0.2341945185

### 3.2 Power control and load scheduling

**Fig 12:**
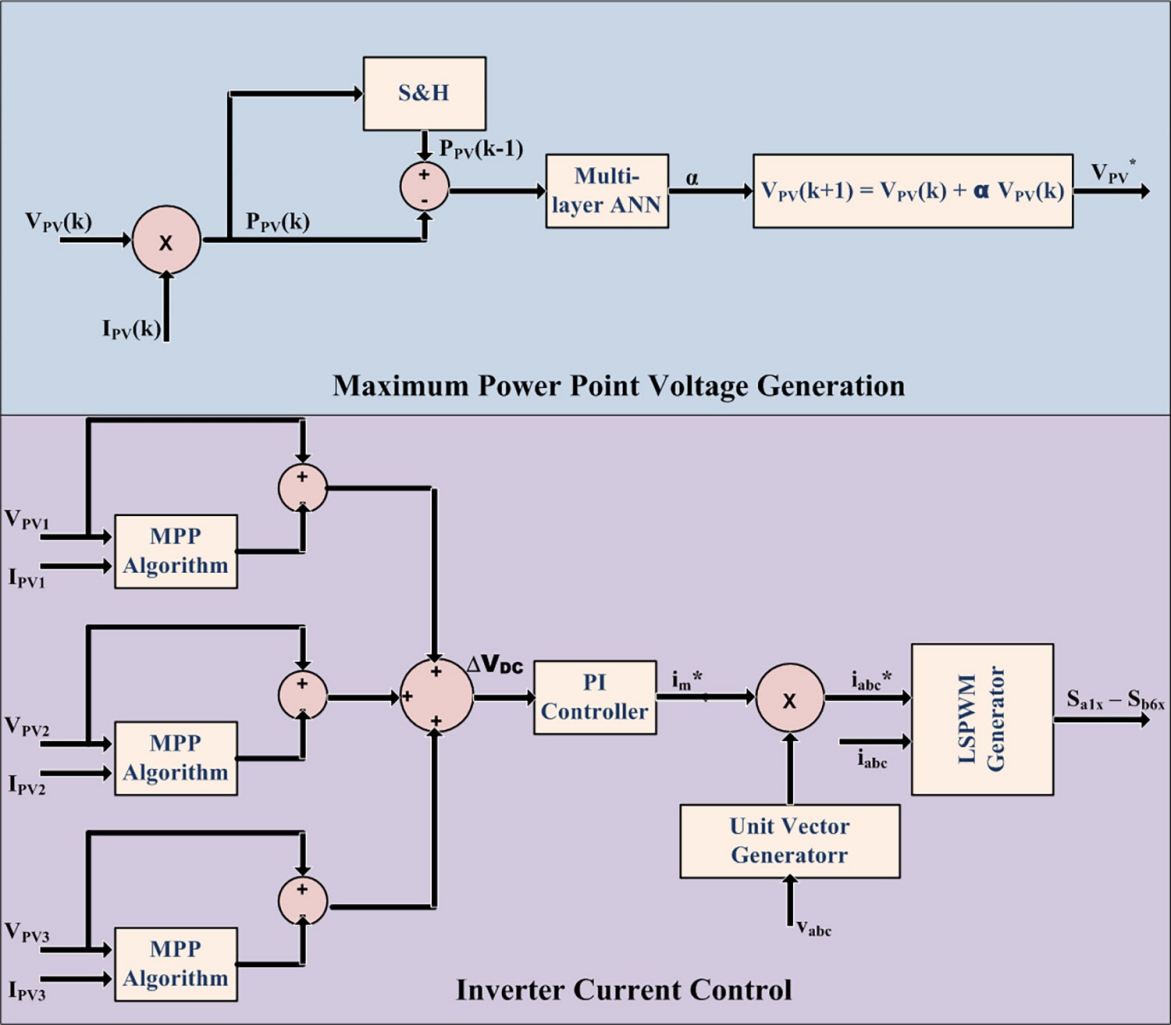
MPP operation and inverter current control.

Voltages and currents of PV arrays are sensed to determine voltage corresponding to maximum power. Control for extracting MPP is shown in Fig 12. The voltage and current are utilized to determine PV array power. The difference in PV power samples along with gradient of PV power fed as input to ANN determines the direction and step size in each iteration to track the maximum power point. Control of single stage modular inverter is also shown in Fig 12. The error in obtained DC voltage from MPP control and actual PV array voltage serve as input to PI controller which determine the modulation ratio of the reference sinusoidal current waveform for regulation of motor phase voltage to set value. The phase reference is obtained from zero crossing instants of respective set phase voltages. The product of these two determines the reference current waveform for each phase. The error in reference and actual currents serve as reference waveform for generating PWM gating signals with level-shifted carries discussed in Section 2.

**Fig 13:**
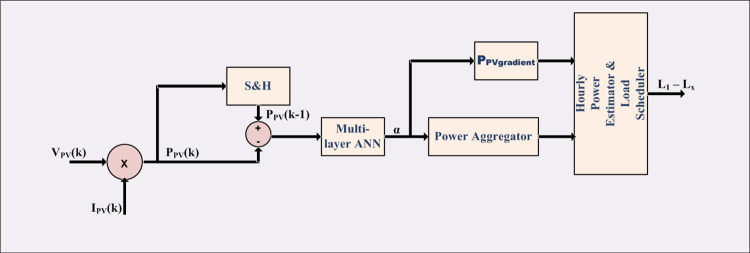
Supervisory load relay control.

The supervisory control of PV power estimation and load relay control are achieved as shown in Fig 13. The gradient is assessed with period of five minutes and PV power is aggregated in every sample. These two inputs are provided to multi-layer ANN with the training pattern similar to that of MPP estimation determines the hourly estimation of PV power. A look-up table then determines the load relay status based on the pre-set load priority.

## 4. Simulation results

The Simulation of inverter with proposed integrated intelligent control is carried out in MATLAB/SIMULINK. The simulation parameters are given in Table 3. The performance is evaluted in terms of efficient power conversion and load schedule. Simulation results for these aspects are presented in this section.

**Table 3:** Simulation parameters.

Parameter	Value
PV Power	1 kW_P_
V_PV1_, V_PV2_, V_PV3_	40 V, 80 V, 240 V
Rated inverter output voltage	230 V (RMS)
Load Power	800 W
Switch Modules	IGBT 600 V, 15 A

### 4.1. Power flow control

**Fig 14:**
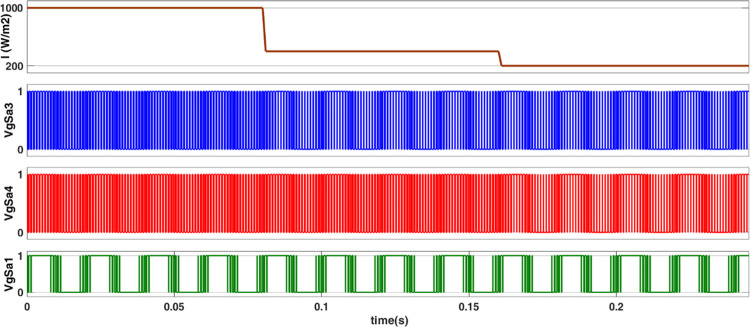
Modulation of converter switches for varying irradiance.

**Fig 15:**
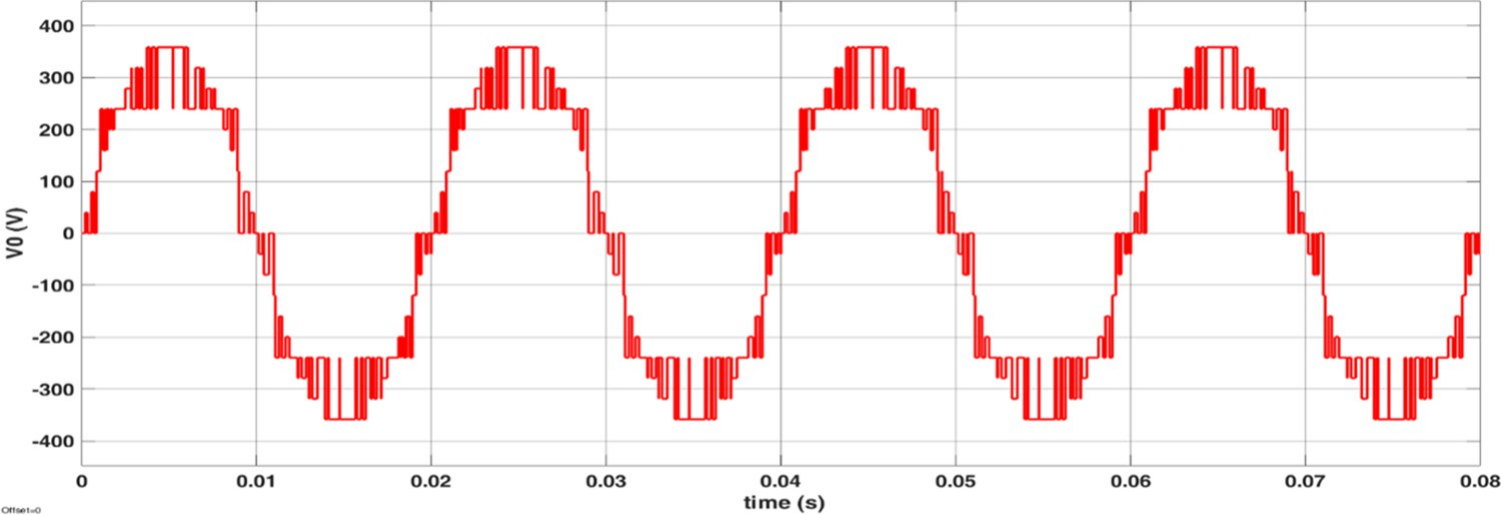
Inverter output voltage.

**Fig 16:**
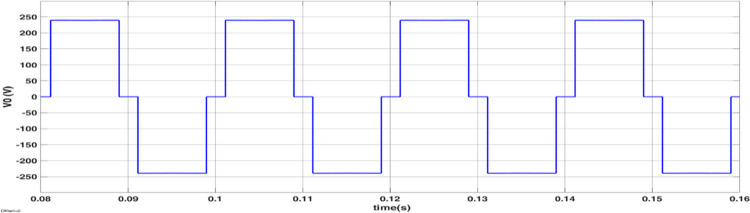
Module 1 output voltage.

**Fig 17:**
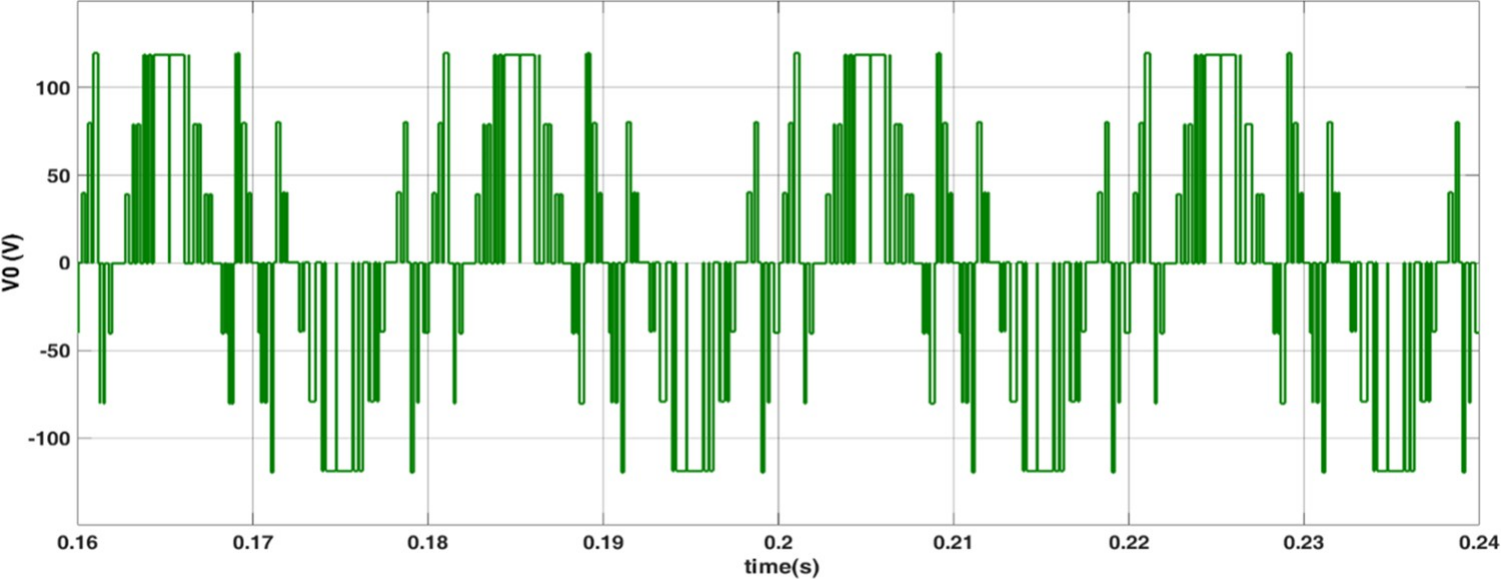
Module 2 output voltage.

**Fig 18:**
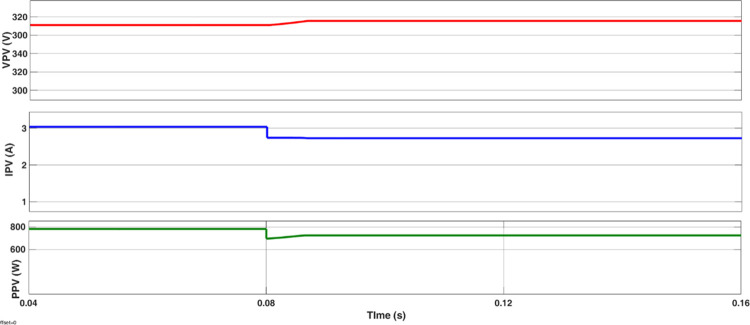
Tracking MPP for change in irradiance.

The simulated output voltage regulation of the inverter is shown in Figs 14–18. Varying irradiance from 1000 W/m^2^ to 400 W/m^2^ at 0.08 sec and to 200 W/m^2^ at 0.16 sec resulted in adjustment of inverter modulation index to regulate output voltage at desired level is seen from Fig 14. The steady state output voltage of the inverter is shown in Fig 15. The contribution of each module for steady state output voltage is shown in Fig 16 and Fig 17 respectively. The robustness of MPP tracking is observed from Fig 18 in which decrement in irradiance at 0.08 sec caused current to drop and voltage to increase to new values as seen from Fig 18 to track new maximum power point corresponding to varied irradiance. The transient time to settle to new steady values is observed to be 0.02 sec.

### 4.2 Load relay control

**Fig 19:**
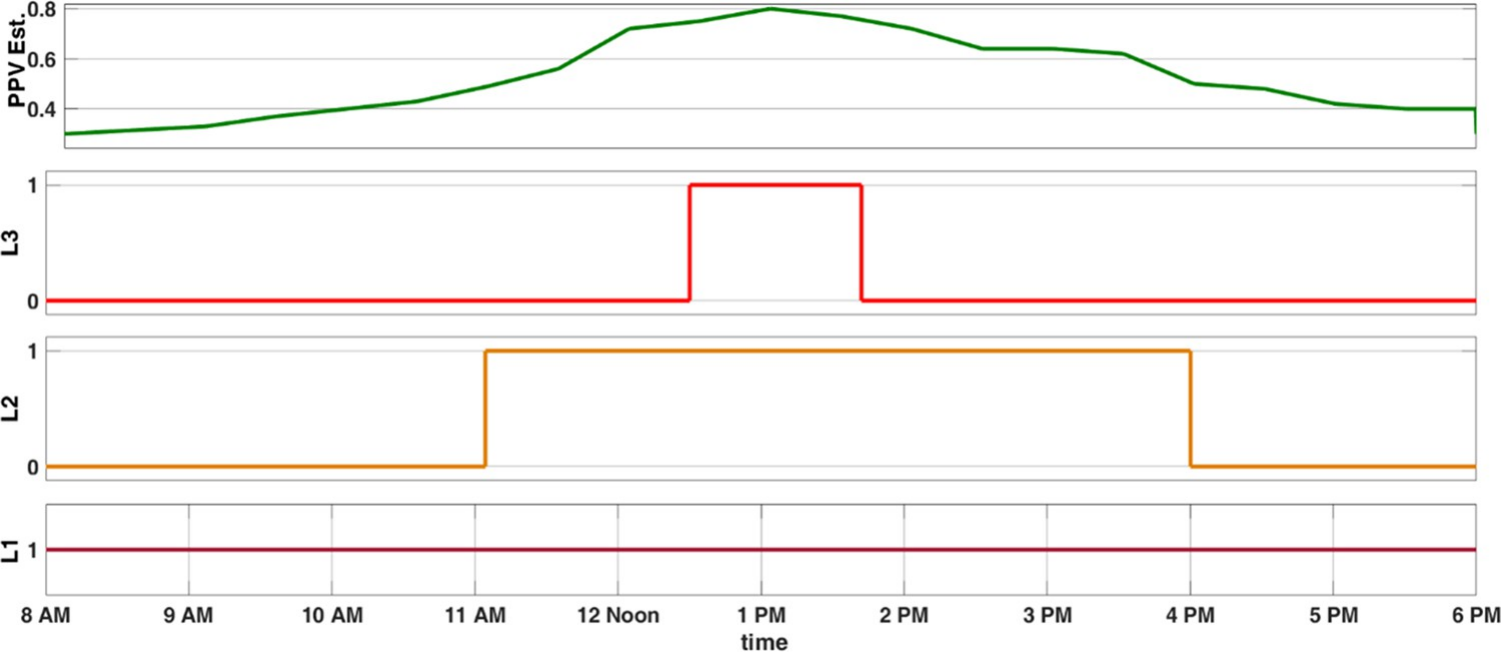
Inverter load schedule for estimated PV power.

The simulated output for load relay control is shown in Fig 19. The estimated PV power from power aggregator and power gradient obtained is shown in Fig 19. Load 1 being the high priority load is always scheduled to be turned ON with minimum power availability of 0.3 pu. Load 2 with the next higher priority is scheduled to turned ON for PV power estimation greater than 0.5 pu. Load 3 with least priority is s scheduled to turned ON for PV power estimation greater than 0.75 pu.

## 5. Experimental validation and performance evaluation

**Fig 20:**
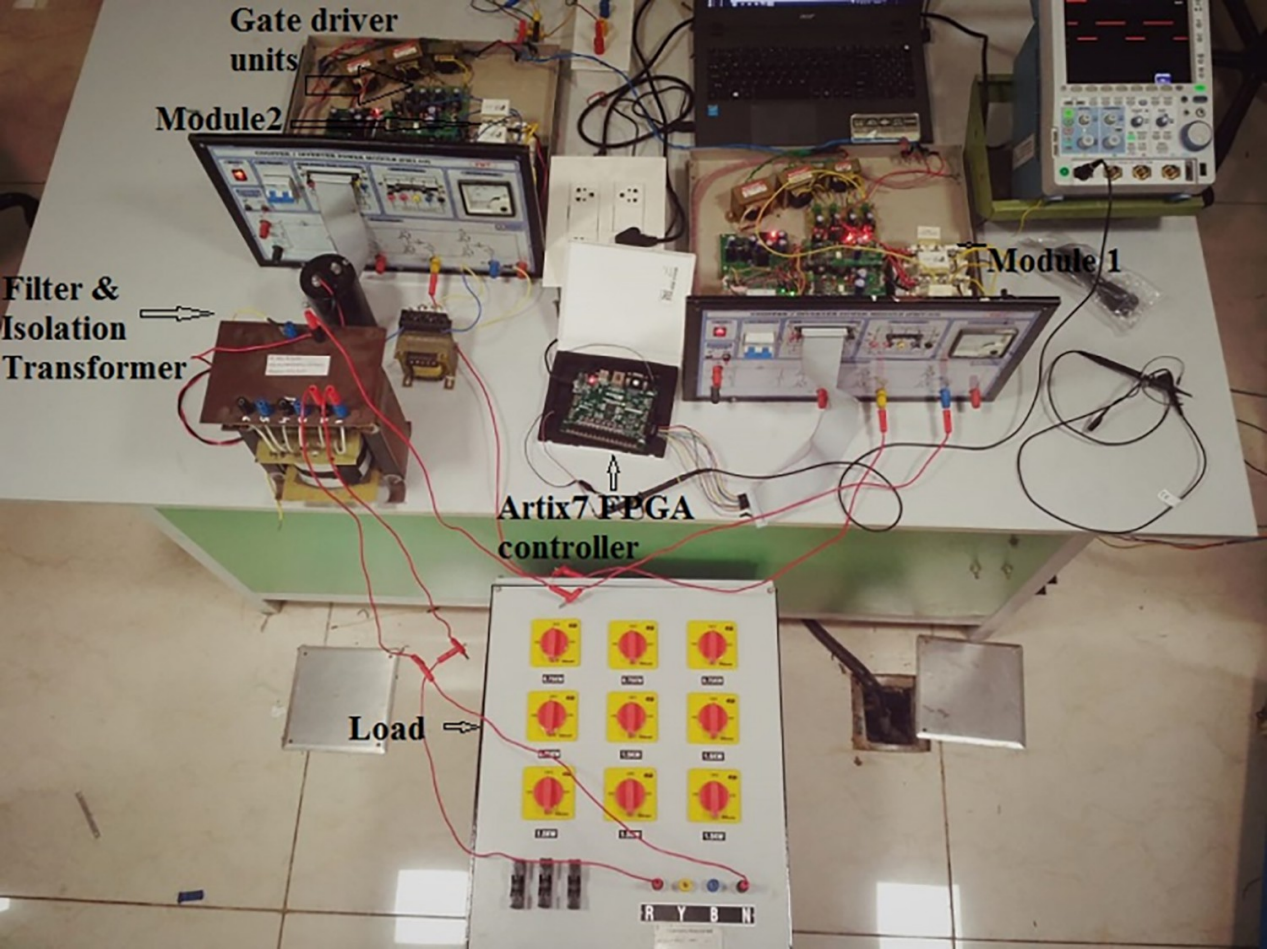
Experimental setup.

A hardware setup is developed to modular inverter. The parameters and hardware modules utilized are provided in Table 4. The experimental setup is shown in Fig 20. The proposed control is realized through Artix 7 FPGA controller.

**Table 4:** Experimental parameters.

Parameter	Nominal Value
Load Power	1 kW
V_DC_	360 V
Power switches	IGBT: FGA25N120AND
Current sensor	LEM LA 25-P, 1000 mA/ 1 mA
Voltage Sensor	LM LV 25-P
Controller	Artix-7 FPGA
Gate Driver unit	TLP 250 based

### 5.1. Power flow control

**Fig 21:**
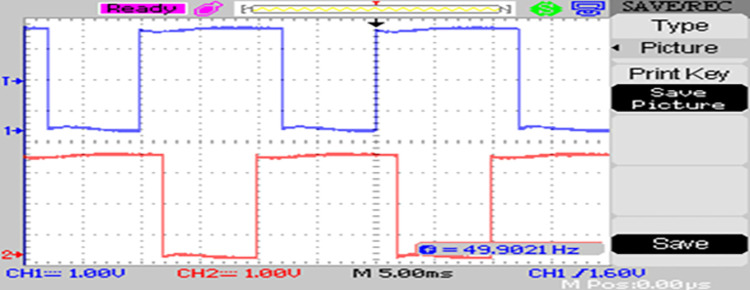
S_a1_, S_a2_ gating signals.

**Fig 22:**
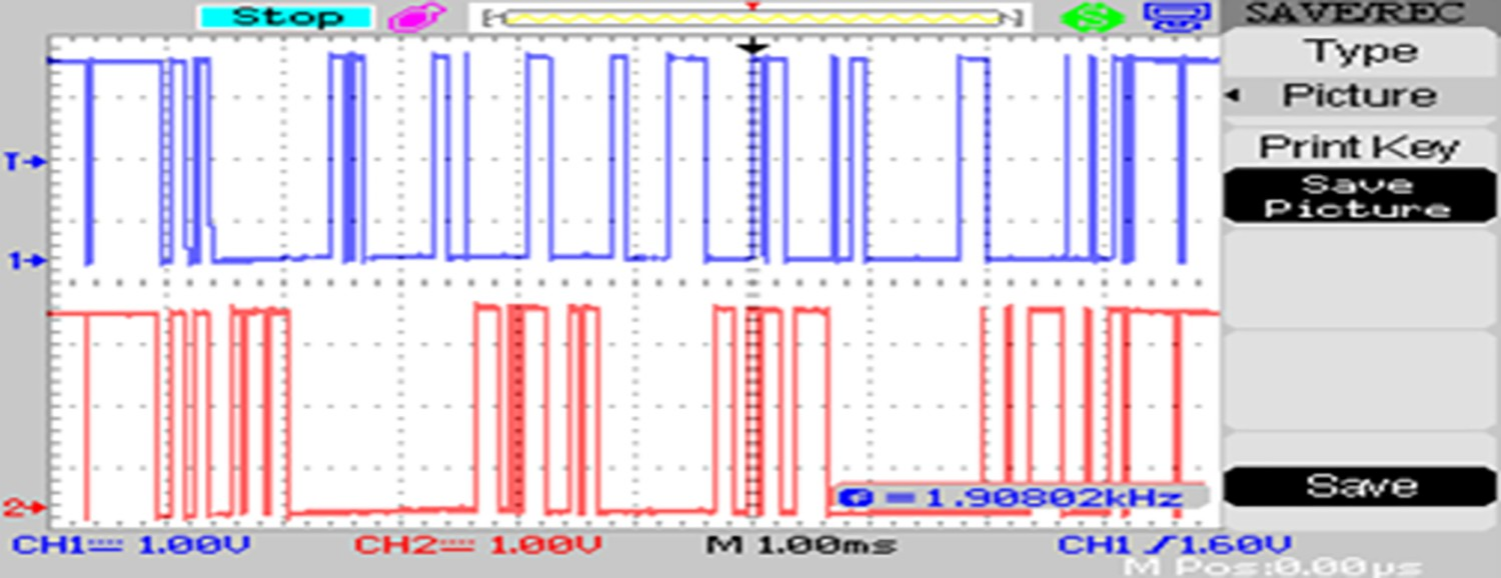
S_a3,_ S_a5_ gating signals.

**Fig 23:**
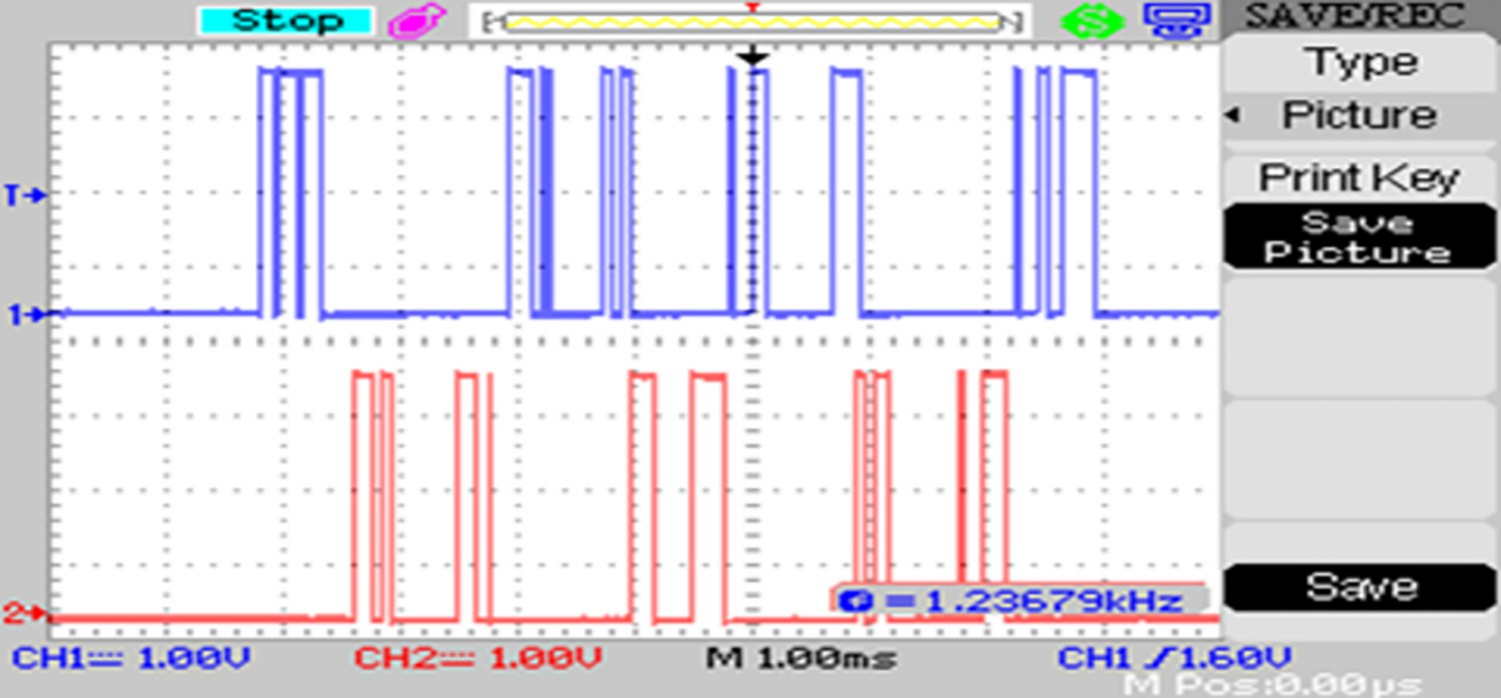
S_b4_, S_b6_ gating signals.

**Fig 24:**
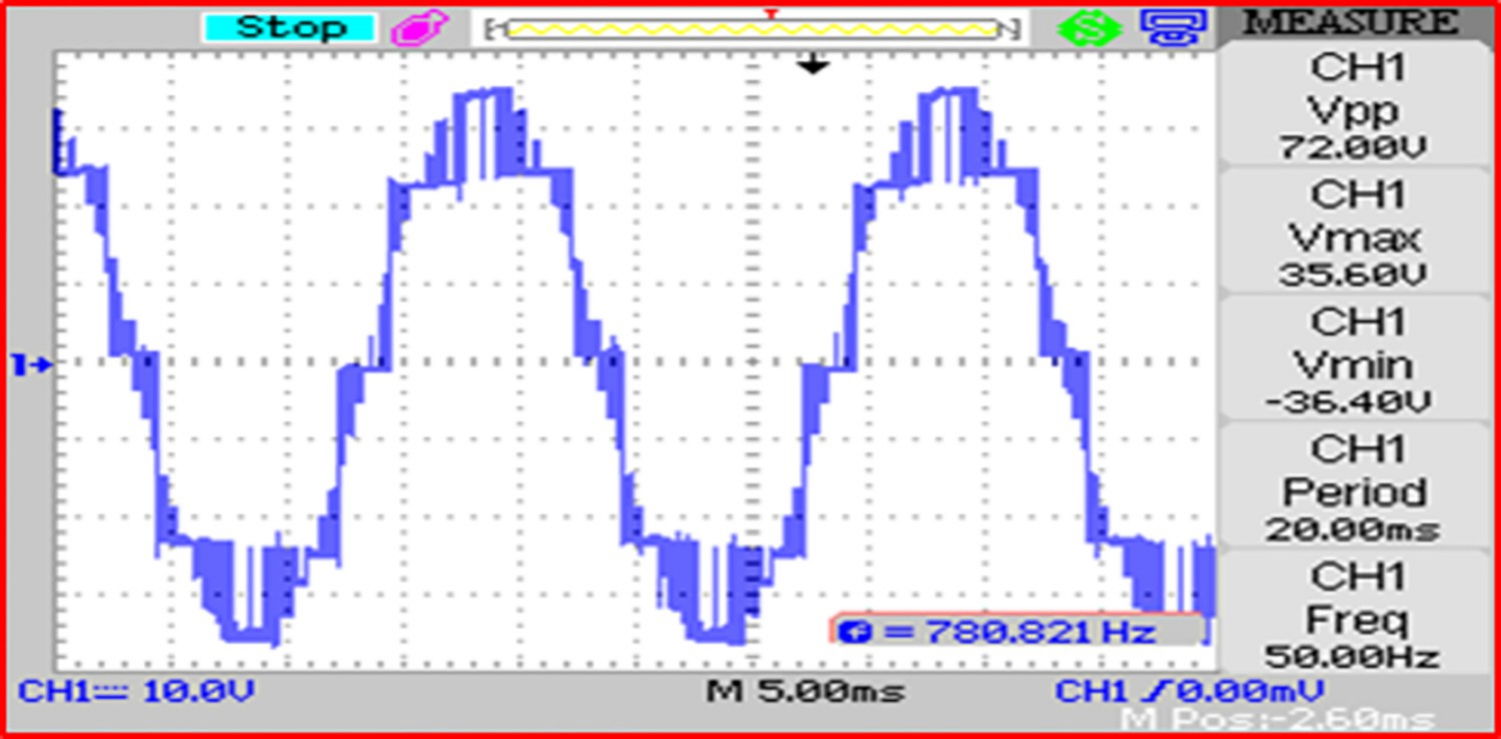
Inverter output voltage (10: 1 attenuation).

**Fig 25:**
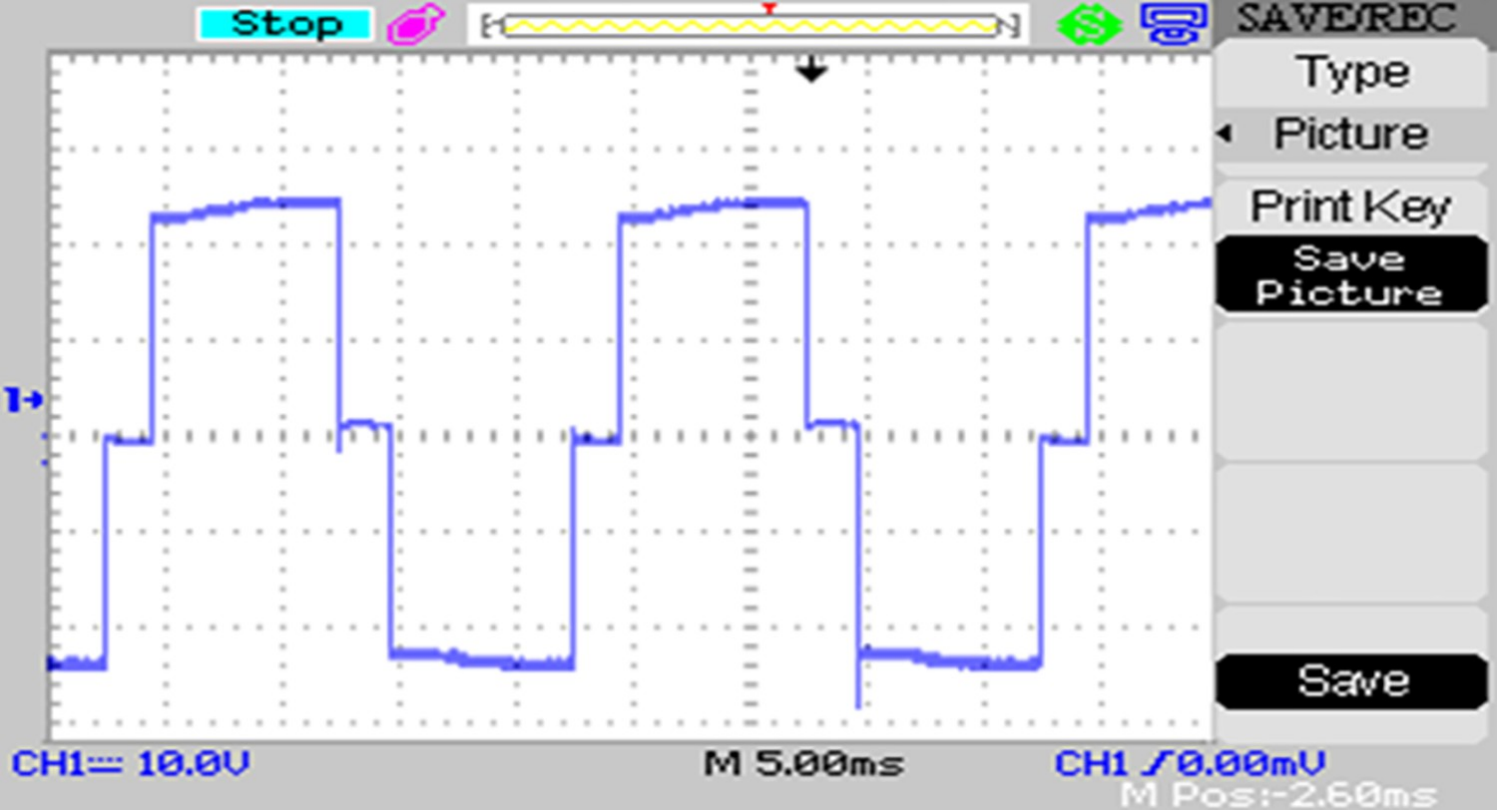
Module 1 output voltage (10: 1 attenuation).

**Fig 26:**
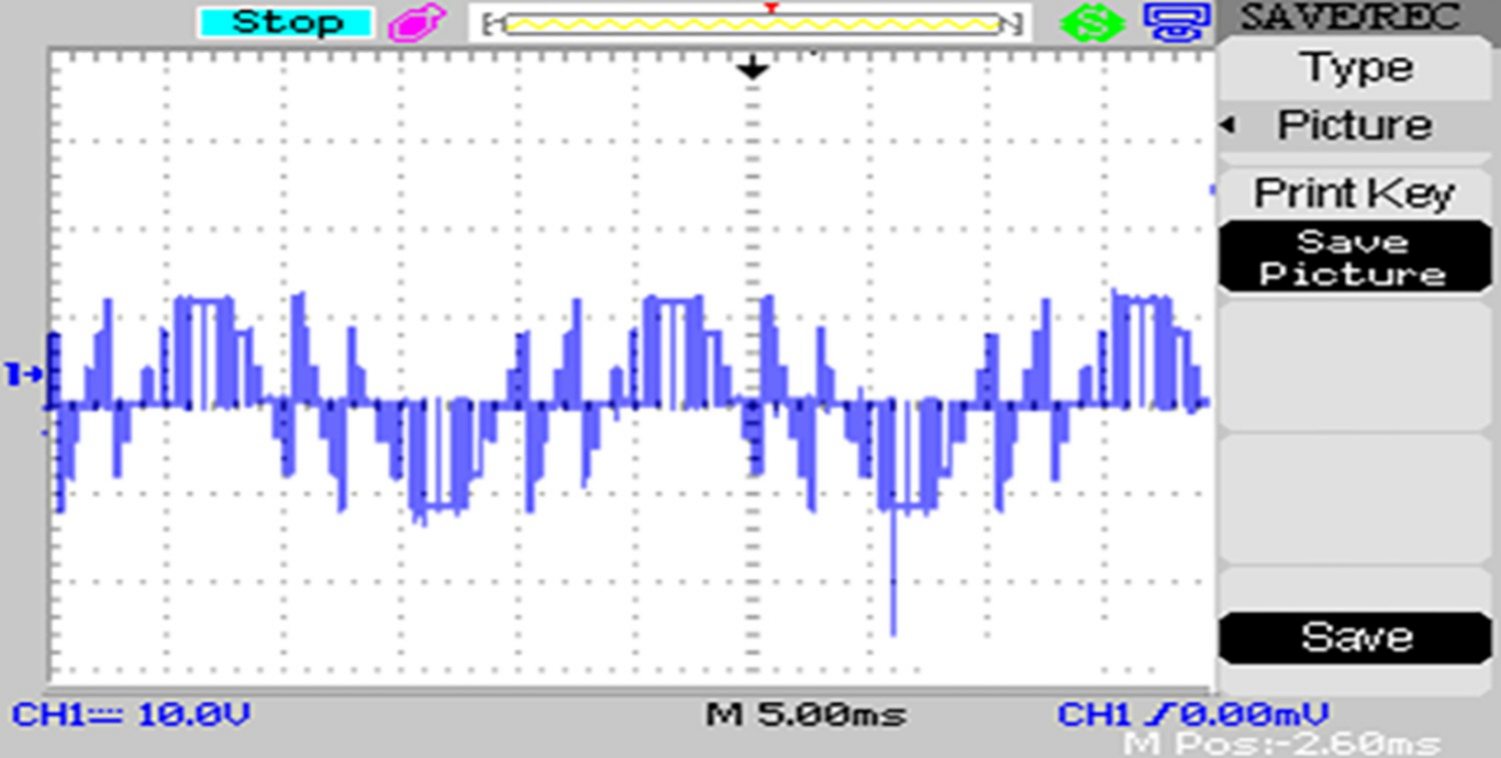
Module 2 output voltage (10: 1 attenuation).

The modulated gating pulses pertaining to instance of irradiance is depicted in Figs 21–23 which corresponds to availability of nominal irradiance. The gating pulse to Module 1 switches is shown in Fig 21. The modulated gating pulses for Module 2 switches is shown in Fig 22 and Fig 23 respectively. The nineteen-level steady output voltage recorded is shown in Fig 24. The THD is obtained to be 18.4 percent. The contributions of Module 1 and Module 2 are shown in Fig 25 and Fig 26 respectively. These validate the simulation waveforms as depicted in section 4.

### 5.2 Load scheduling

**Fig 27:**
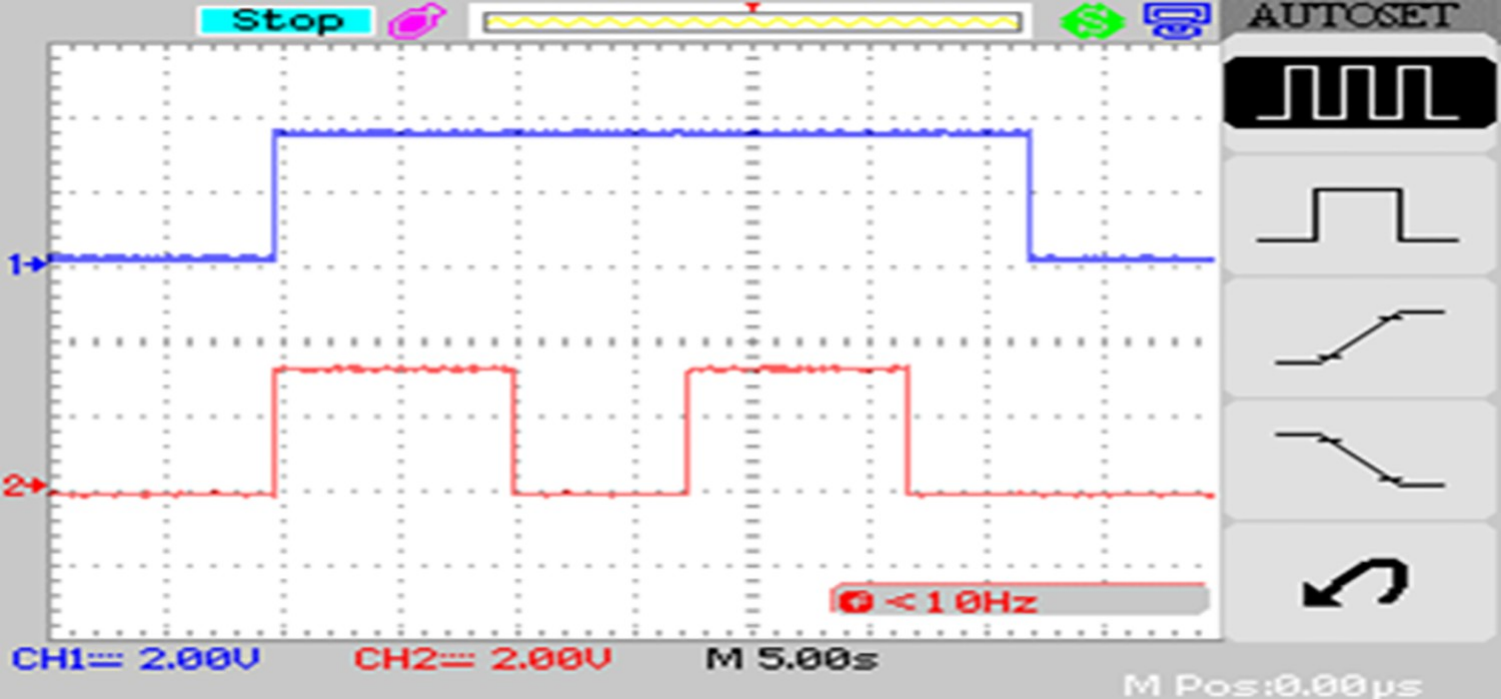
Load relay control for estimated PV power.

The estimated PV power from power aggregator and power gradient control the load relays which are scheduled to turned ON for PV power estimation greater than 0.5 pu for Load 2 and greater than 0.75 pu for Load 3 which is seen from Fig 27.

### 5.3 Converter sizing and reliability evaluation

The rms voltage and current ratings are measured and per unit voltage and current sizing is provided in Table 5.

**Table 5:** Power switch sizing.

Switches	RMS current (pu)	Average Conduction Power loss(pu)	Voltage Rating (pu)
S_a1_,S_a1’_,S_a2_, S_a2’_	0.57	0.005	0.67
S_b1_,S_b3_,S_b1’_, S_b2_,S_b2’_,S_b4_	0.48	0.0046	0.33
S_b5_,S_b6_	0.24	0.0005	0.33

**Fig 28:**
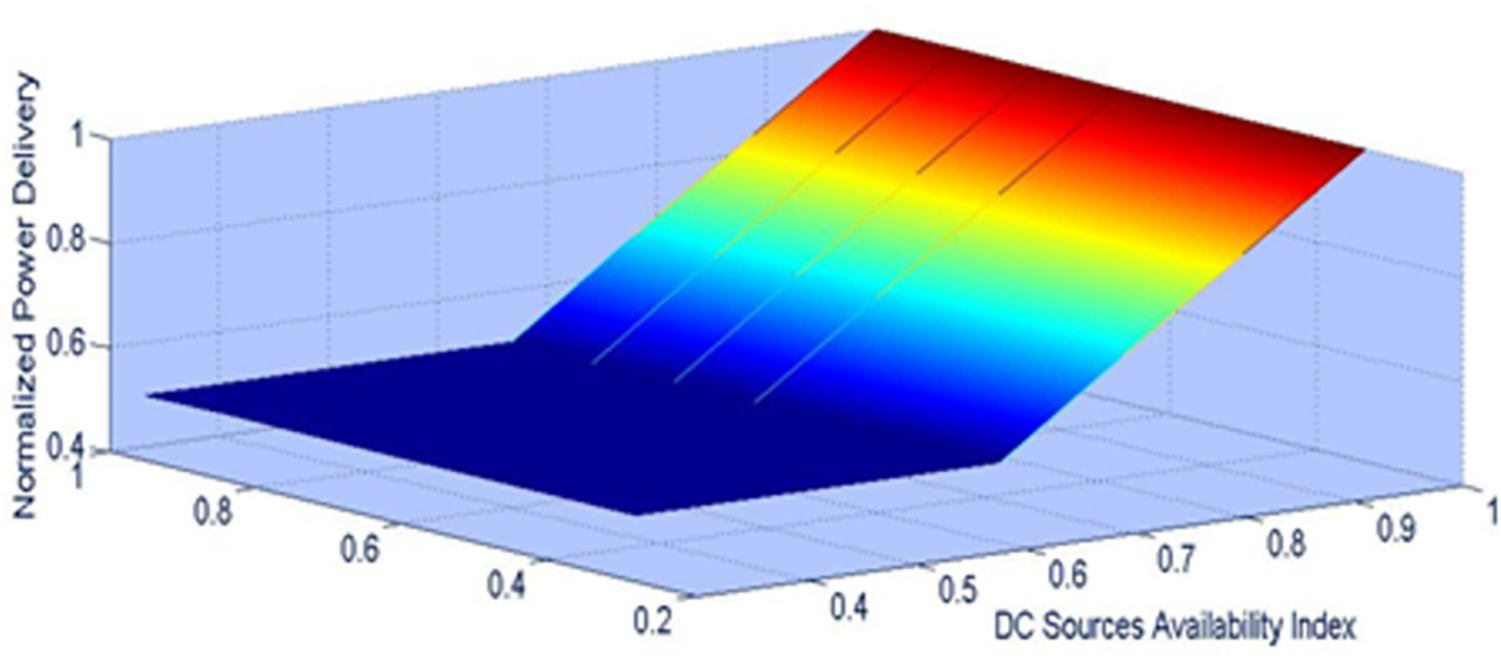
Normalized power delivery for dc source failure.

**Fig 29:**
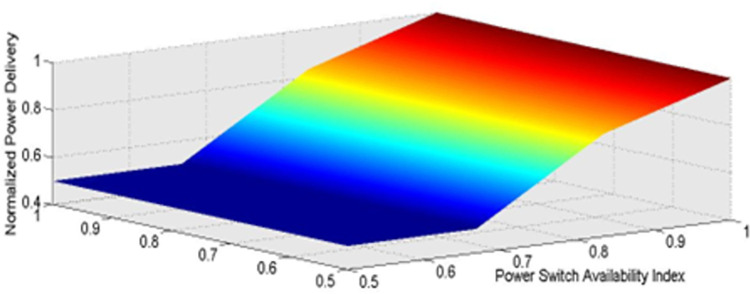
Normalized power delivery for switch failure.

**Fig 30:**
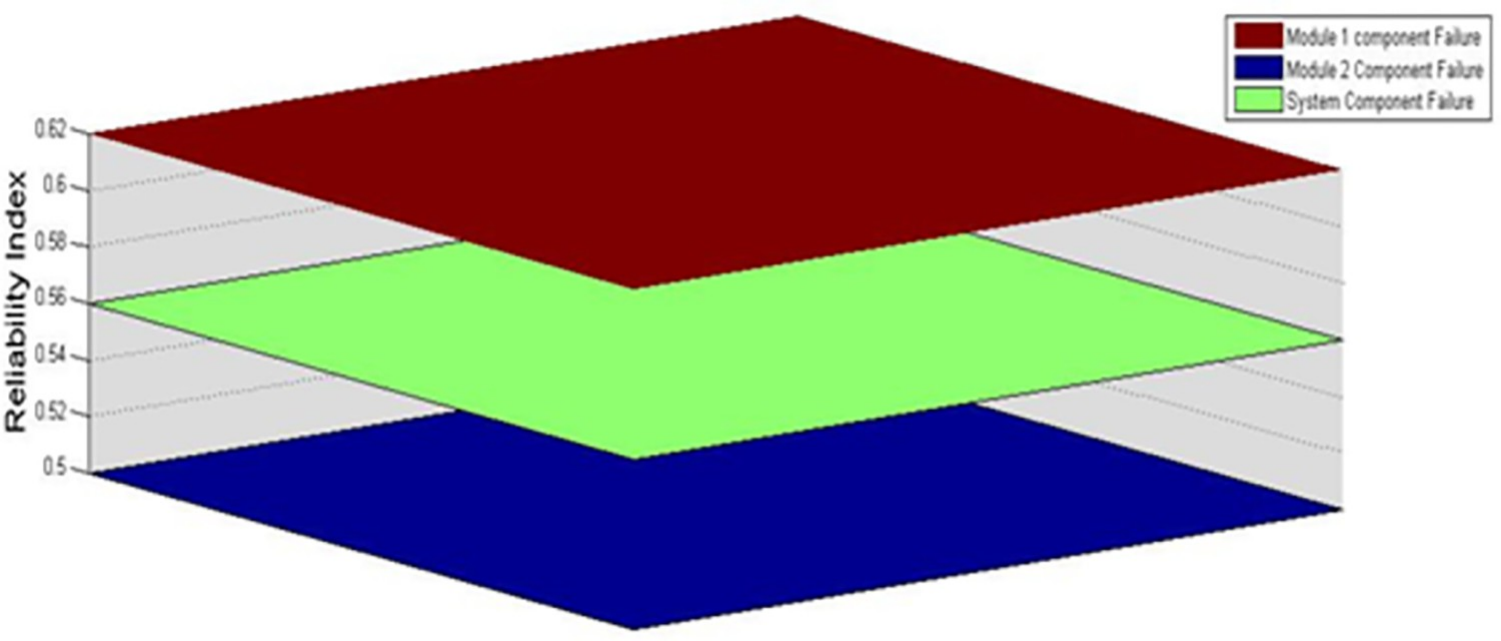
Reliability factor for proposed topology.

Another figure of merit for the proposed control is that utilizing modular nature of the converter the modulation index obtained from supervisory control determines the output levels of inverter voltage. The power transferring capability of the inverter upon sources failure or switch failure is determined as shown in Fig 28 and Fig 29 respectively where in half of the rated power could be transferred in worst case. The power delivery for module 1 component failures and module 2 component failures is shown in Fig 30. The average power delivery upon any component failure which is determined to be 56 percent.

A comparison is made in terms of minimum output power delivered by the inverter under module or source failure is presented in Table 6. The proposed inverter with its topological structure proved minimum of 56 percent power availability for any component failure in comparison to existing similar switch count asymmetrical multilevel inverters.

**Table 6:** Comparison of redundancy.

Topology	Minimum per unit output power upon component Failure
Proposed	0.56
[12]	0.5
[19]	0.33
[16]	0.25
[22]	0.25

A comparison among the existing and proposed modular inverters is made in terms of number of levels in output voltage, and sizing of power switches. The proposed bi-modular achieved more than 50 percent reduction in voltage and current sizing compared to conventional H-bridge [26] inverters with similar switch count. Also, Table 7 depicts the comparison of sizing with asymmetrical inverters in which the proposed converter proved reduced size with existing asymmetrical inverters.

**Table 7:** Comparison of modular inverters.

Twelve switch topologies	Output voltage level generation	Average PU Switch Voltage Rating	Average PU Switch Current Rating
Proposed	19	0.44	0.43
[12]	7	0.33	1
[16]	7	0.5	1
Conventional MLI [26]	7	1	1

## 6. Conclusions

A bi-modular nineteen-level PWM voltage source inverter is developed for high-power applications. The proposed inverter is controlled with machine learning-based control for extracting MPP, inverter power control and PV power estimation. The MPP extraction is achieved with 99.9 percent accuracy. The machine learning algorithms accurately determine the modulation of inverter voltage for changing MPP. The presented twelve-switch modular inverter validated a nineteen-level output voltage 2.7 times higher than conventional similar switch count topologies. The performance of the proposed inverter is proved compatible over reasonable power factor range and modulation range. Also, a reduction by 20 percent is obtained in terms of power switch sizing compared to similar power handling conventional topologies. The reliability study proved its redundancy by 56 percent. Thus, this inverter topology and control algorithm can be implemented for high power applications for efficient and reliable power electronic interfaces.

## Supporting information

S1 File Training data1. (XLSX)

S2 File Training data2. (XLSX)

S3 File Training data3. (XLSX)

S4 File Training data4. (XLSX)
